# Phenotypic differences between highlanders and lowlanders in Papua New Guinea

**DOI:** 10.1371/journal.pone.0253921

**Published:** 2021-07-21

**Authors:** Mathilde André, Nicolas Brucato, Sébastien Plutniak, Jason Kariwiga, John Muke, Adeline Morez, Matthew Leavesley, Mayukh Mondal, François-Xavier Ricaut

**Affiliations:** 1 Estonian Biocentre, Institute of Genomics, University of Tartu, Tartu, Tartumaa, Estonia; 2 Laboratoire Évolution and Diversité Biologique (EDB UMR5174), Université de Toulouse Midi-Pyrénées, CNRS, IRD, UPS, Toulouse, France; 3 Laboratoire Travaux et Recherches Archéologiques sur les Cultures, les Espaces et les Sociétés (TRACES, UMR 5608), Université Toulouse Jean Jaurès, Maison de la Recherche, Toulouse, France; 4 Strand of Anthropology, Sociology and Archaeology, School of Humanities & Social Sciences, University of Papua New Guinea, National Capital District, Papua New Guinea; 5 School of Social Science, University of Queensland, Australia, St Lucia, Australia; 6 Social Research Institute Ltd, Port Moresby, Papua New Guinea; 7 School of Biological and Environmental Sciences, Liverpool John Moores University, Liverpool, United Kingdom; 8 ARC Centre of Excellence for Australian Biodiversity and Heritage, College of Arts, Society and Education, James Cook University, Cairns, Australia; Universitat Pompeu Fabra, SPAIN

## Abstract

**Objectives:**

Altitude is one of the most demanding environmental pressures for human populations. Highlanders from Asia, America and Africa have been shown to exhibit different biological adaptations, but Oceanian populations remain understudied [Woolcock et al., 1972; Cotes et al., 1974; Senn et al., 2010]. We tested the hypothesis that highlanders phenotypically differ from lowlanders in Papua New Guinea, as a result of inhabiting the highest mountains in Oceania for at least 20,000 years.

**Materials and methods:**

We collected data for 13 different phenotypes related to altitude for 162 Papua New Guineans living at high altitude (Mont Wilhelm, 2,300–2,700 m above sea level (a.s.l.) and low altitude (Daru, <100m a.s.l.). Multilinear regressions were performed to detect differences between highlanders and lowlanders for phenotypic measurements related to body proportions, pulmonary function, and the circulatory system.

**Results:**

Six phenotypes were significantly different between Papua New Guinean highlanders and lowlanders. Highlanders show shorter height (p-value = 0.001), smaller waist circumference (p-value = 0.002), larger Forced Vital Capacity (FVC) (p-value = 0.008), larger maximal (p-value = 3.20e -4) and minimal chest depth (p-value = 2.37e -5) and higher haemoglobin concentration (p-value = 3.36e -4).

**Discussion:**

Our study reports specific phenotypes in Papua New Guinean highlanders potentially related to altitude adaptation. Similar to other human groups adapted to high altitude, the evolutionary history of Papua New Guineans appears to have also followed an adaptive biological strategy for altitude.

## Introduction

New Guinea was settled 49,000 years ago (kya) [1] making it one of the oldest continuous human occupations outside Africa. This territory is characterised by diverse environments, from the large coastal lowlands to the highlands which host the highest mountains in Oceania [2, 3]. New Guinean populations initiated their settlement in the lowlands and established permanent settlements in the highlands 20 kya [1, 4–7]. New Guinean highlanders remained relatively genetically isolated through time with only minor external influences [5, 8]. For thousands of years, New Guinean highlanders and lowlanders followed different cultural trajectories. The independent emergence of agricultural practices around 9 kya in the highlands favoured the demographic expansion of these groups. On the contrary, agricultural practices were brought to the lowlands through the Austronesian influence around 3 kya [9]. Currently, the Papua New Guinea (PNG) highlands is the most densely populated area of the country, representing almost 40% of the total population (so approximately 3 million highlander individuals) [10]. Many of these groups live at an altitude ranging between 1,400 and 2,850 m above sea level (a.s.l.) [11]. PNG highlanders and lowlanders therefore differ, not only in their history, but also in their living environment. These two aspects could have influenced the physical characteristics of these populations. In this paper, we concentrate on phenotypic differences related to altitude.

Altitude is one of the strongest environmental stresses for human populations [12]. At high altitude, the partial pressure of oxygen drops due to low air pressure which ultimately decreases the oxygen supply to human tissues [13, 14]. At the altitude limit of 2,500 m a.s.l., which generally defines high-altitude, physiological effects are clearly detectable because the partial pressure of oxygen is around 70% of its level at sea level [13]. When ascending to high altitude, people from lower altitudes experience an acute response: hyperventilation [14, 15], increased heart rate [14, 16], and high blood pressure [16–18]. If these individuals remain at high altitude, they may undergo a chronic response and the number of their red blood cells increases to accommodate the hypoxic conditions [14, 15].

Despite these detrimental conditions, some human populations have permanently settled in high-altitude locations. For thousands of years, Tibetans, Andeans, and Ethiopian Amharas have lived at high altitude, which has led to the emergence of specialised cultures and an adapted biology [19]. These populations have phenotypic traits that appear to be influenced by hypoxia at high altitude [20]. Andeans exhibit a broader chest, larger lung capacity and higher haemoglobin concentration compared with lowlanders [21–23]. Tibetans show lower haemoglobin concentrations due to higher plasma volumes [23, 24]. The moderate increase in haemoglobin concentration [25–27] and an enlarged chest size [28] in Ethiopian highlanders can be comparable to what is observed among Andeans, albeit not being to the same extent. However, this increased haemoglobin concentration in Ethiopians has not been replicated by all studies [29–31]. These traits may also be the result of other non-mutually exclusive factors [32]: cold temperatures at altitude [33], genetic drift [34], high-altitude exposure during growth [35], lifestyle [13, 36], and socioeconomic status [37].

Despite the complex nature of phenotypic traits usually related to high-altitude adaptations [38], genomic analyses have shown that many traits are partly coded by genes under selective pressure, especially by genes of the Hypoxia-Inducible Factor (HIF) pathway [39]. Variants of HIF-2α, known as EPAS1, were associated with lower haemoglobin concentrations [40, 41]. EPAS1 variants in Tibetan genomes are particularly noteworthy as they seem to be introgressed from Denisovans [42]. Selection signals were also detected in other genes of the HIF pathway, such as EGLN1 in Andeans and Tibetans [39], and BHLHE41 in Ethiopian highlanders [30].

Previous studies on the health of PNG populations have shown that highlander groups have some specific phenotypic variations potentially linked to altitude: an increase in ventilatory lung function and haemoglobin concentration [43–45]. While these data were not specifically studied in relation to high altitude adaptation, they strongly suggest that PNG highlanders could display similar phenotypic traits as those observed in other high-altitude populations worldwide [43–45].

In this study, we tested the hypothesis that PNG highlanders have developed phenotypic traits known to be adapted to altitude, as a result of inhabiting the highest mountains in Oceania for at least 20 kya. We collected data on 13 phenotypic traits related to altitude for 162 PNG individuals living at high and low altitude locations. We explored the variability of these phenotypes to: (i) identify phenotypic traits specific to PNG highlanders, and (ii) compare this variation to other high-altitude human groups.

## Methods

### Ethics

This study was approved by the Medical Research Advisory Committee of Papua New Guinea under research ethics clearance MRAC 16.21 and the French Ethics Committees (Committees of Protection of Persons IE-2015-837 (1)). Permission to conduct research in Papua New Guinea was granted by the National Research Institute (visa n°99902292358) with full support from the School of Humanities and Social Sciences, University of Papua New Guinea.

### Sampling

**Fig 1:**
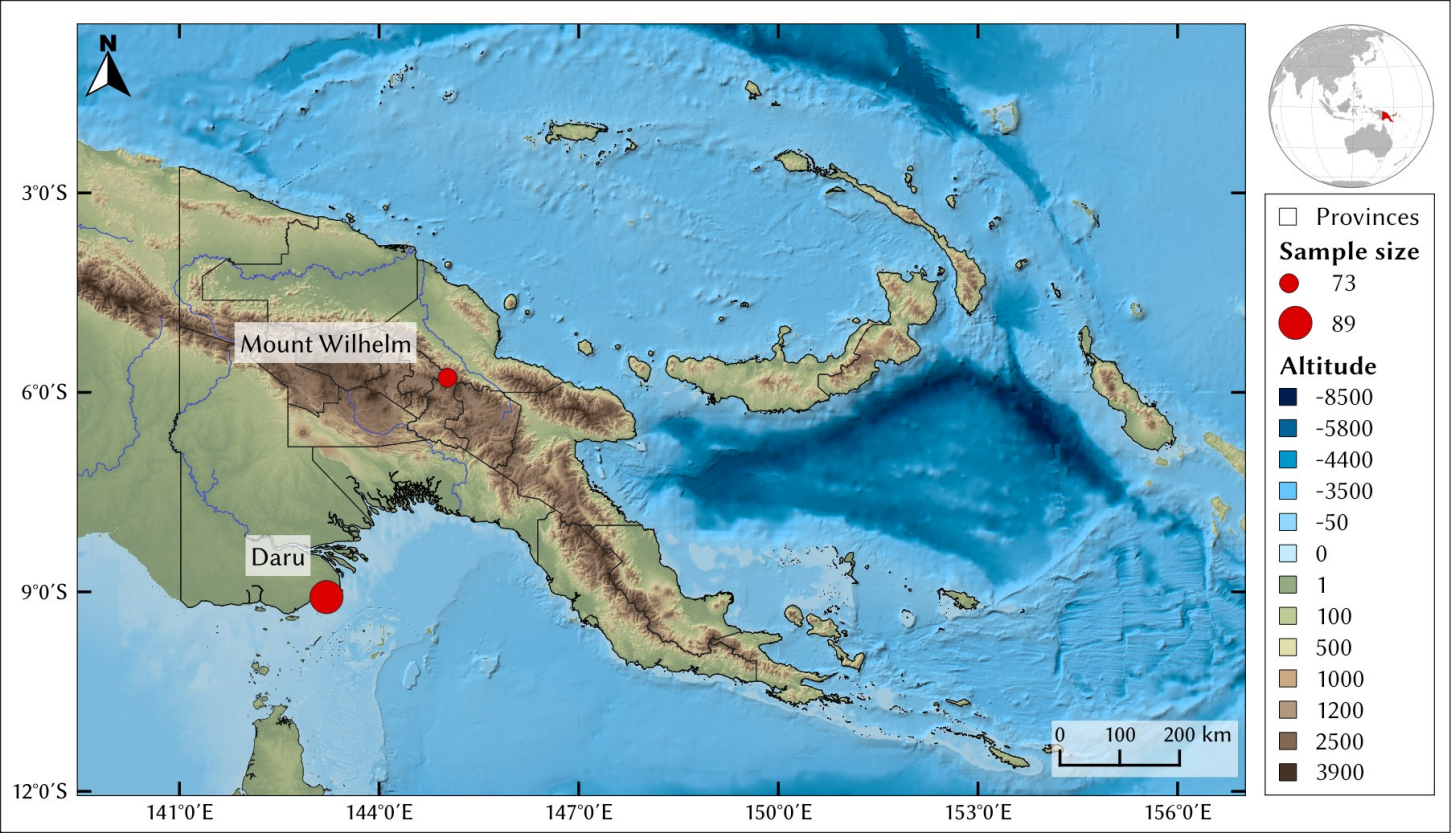
Map of the sampling locations. Map: Sébastien Plutniak/QGIS 3.14.16. Projection: EPSG 4326. DOI: 10.5281/zenodo.3911844. Sources: digital elevation model: GEBCO [46].

All data were collected from unrelated healthy adults (42.19 years old [95% CI 39.66–44.72]) after they had signed informed consent forms. None of the women participating in the study were pregnant. A presentation of the project was shown to all participants, followed by a discussion with each person to ensure that they had understood the project. Participants completed a questionnaire to determine language affiliation(s), current residence, date and place of birth, and a short genealogy of up to two generations to establish regional ancestry. We excluded individuals who had one or more parents or grandparents from another region (e.g. province) and only kept individuals with a full regional ancestry over at least two generations. Phenotypic data were collected from a total of 162 individuals by the same investigators using the same equipment between November 2016 and August 2019 in two different places (Fig 1). Data was collected from 73 highlander individuals in the villages of Womatne (2,300 m a.s.l.), Gembogl (2,500 m a.s.l.) and Keglsugl (2,700 m a.s.l.), located on the slope of Mount Wilhelm (Chimbu Province) (Fig 1). Populations from this region were assumed to have no significant Asian genetic input because they have been genetically separated from PNG lowlanders since at least 10 kya [5, 8]. The territory of these highlander populations extends to high altitude for gardening (up to the frost line around 2,800 m a.s.l.) and even higher for hunting [11]. No short- or long-term stays in lower altitude locations were reported by the participants. Three individuals from Mount Wilhelm were excluded from our analyses because at least one of their parents and/or grandparents did not originate from the PNG highlands, resulting in a final sample size of 70 highlander individuals. For the lowland sampling campaign, data were collected from 89 individuals living below 100 m a.s.l. in Daru (Western Province) (Fig 1). We excluded three of these individuals from further analyses because at least one of their parents and/or grandparents originated from outside the PNG lowlands. All our analyses are based on data collected for 70 PNG highlanders from Mount Wilhelm, and 86 PNG lowlanders from Daru.

### Phenotypic data collection

We measured phenotypic traits reported as adaptive in other highlander groups worldwide [15, 47], allowing us to compare our results with previous work on other highlander populations.

The studied phenotypes correspond to three categories: body proportions, pulmonary function and circulatory system. Measurements were explained to participants in detail. Participants were at rest during data collection, and without shoes. Measurements were taken by a member of the same group of researchers who had been trained in the standardised protocols. Sample sizes were not necessarily the same for each phenotypic measurement (S1 Table).

Height was measured with a rigid ruler following the standard protocol [48]. The waist circumference was measured at the umbilicus [49] using the SECA 201 circumference measuring tape (Hamburg, Germany) [50]. These measurements were converted to the nearest centimetre. Bodyweight was measured with a precision of 100g using a SECA electronic scale. The body-mass index (BMI) was calculated as weight (kg) divided by squared height (m^2^).

Minimal and maximal chest depth was measured with an anthropometer (Lafayette Large anthropometer, Holtain Ltd, Harpenden, UK), after maximal expiration and maximal inspiration, respectively, at the height of the fifth thoracic vertebra [51]. A portable spirometer (Spirobank II, Medisav Ltd, Dorset, UK), with a disposable turbine (Turbine FLOWMIR, MIR), was used to estimate pulmonary function [52]. We measured: the volume of air that is forcibly blown out in the first second after full inspiration (forced expiratory volume in 1 second, FEV1), the maximal flow achieved during the maximal forced expiration initiated at full inspiration (peak expiratory flow, PEF) and the volume of air that is forcibly blown out after full aspiration (forced vital capacity, FVC) [52]. Each participant performed the test 2–3 times. Winspiropro (8.2.0) software was used to visualise the flow-volume curves. We excluded the curves with excessive extrapolated volume, with no peak (caused by a sub-maximal blast) or several peaks (caused by extra breaths or coughs) [53]. When several flow curves were acceptable for one participant, we chose the best curve according to the software [54].

Haemoglobin concentration was measured using a portable haemoglobin analyser (Diaspect TM and haemoglobin cuvettes, EKF, France) [55]. Blood pressure was measured with a blood pressure monitor (Omron RS3, Omron, Mannheim, Germany) [56] on the left wrist. Heart rate was measured using the same blood pressure monitor and/or a Nonin 8500 pulse oximeter (Nonin Medical, Plymouth, MN) attached to the left index finger (depending on the participant). The heart rate value, measured in beats per minute (bpm), was calculated using the mean of the two measurements.

### Statistical analysis

The mean of each phenotypic measurement in PNG highlanders and lowlanders was compared with a Mann-Whitney U test [57]. We modelled the relationship between each phenotype, age and sex with a multilinear regression in R to correct phenotypic impact by age and sex [57]. We subtracted the predicted value *y*_*i*_—obtained by regression (Eq 1)—from the initial observed data (Eq 2). The residual results of these subtractions were used to test whether there is a difference between the two groups (highlanders and lowlanders) for each phenotype, controlling for the effects of age and sex. As height could be a covariable to other phenotypes, we also regressed height for chest depth [21, 58], waist circumference [59], weight [60], and pulmonary function measurements (FEV1, PEF and FVC) [52, 61–63].


$$y_{i}=\beta_{0}+\beta_{1}{\mathrm{Age}}_{i}+\beta_{2}{\mathrm{Gender}}_{i}(+\beta_{3}{\mathrm{Height}}_{i})+\varepsilon_{i}$$ (1)

Residual=observedphenotype‐predictedphenotype (2)

The analysed phenotypes clustered into five groups of correlated phenotypes (S1 Fig). Mann-Whitney U tests were considered as significant if the p-value was lower than 0.05 after Bonferroni correction for five multiple tests (adjusted p-value = 0.01). We built 95% confidence intervals for each mean by bootstrapping the same number of samples from the dataset with a replacement, to obtain 10,000 bootstrap samples for each phenotype. Then, we used quantiles of the mean samples to obtain the 95% confidence interval (CI, 0.025 and 0.975 quantiles) for each phenotype. In some cases, the Mann-Whitney U test assumption of a continuous distribution without equal values was not met. In these cases (pulmonary function measurements and haemoglobin concentration) we added small independent noise by using jitter in R with the default values [57, 64, 65]. All plots were obtained using R [57, 66]. A map of the sampling locations was generated with QGIS [67].

## Results and discussion

This study describes the phenotypic differences between highlanders and lowlanders in PNG (Fig 1). Among the 13 studied phenotypes linked to high-altitude adaptation (S2 Table), six phenotypes are significantly different between these two groups: height, waist circumference, Forced Vital Capacity (FVC), minimal and maximal chest depth, and haemoglobin concentration (Table 1, S2–S9 Figs). They reflect a combination of phenotypic adaptations specific to high altitude in Oceania, complementing the well-studied cases from Asia (Tibetans, [42, 68]), America (Andeans [52]) and Africa (Ethiopians, [30]).

**Table 1:** Phenotypic comparison between PNG highlanders and lowlanders.

	PNG lowlanders	PNG highlanders	Mann-Whitney U test		
	N = 86	N = 70			
	Mean [95% CI]	Mean [95% CI]	raw data^a^	Residuals age, sex^b^	Residuals age, sex, height^c^
*Body proportions*					
**Height (cm)**	**168.8**	**164.6**	5.15e -4*	0.001*	-
	**[166.84–170.72]**	**[163.21–166.03]**			
Weight (kg)	68.46	64.82	0.137	0.224	0.873
	[65.86–71.15]	[63.21–66.53]			
BMI (m^2^/kg)	24.04	24.00	0.661	0.337	-
	[23.19–24.97]	[23.33–24.76]			
**Waist circumference (cm)**	**91.77**	**83.54**	3.50e -7*	5.26e -6*	0.002*
	**[89.44–94.16]**	**[81.99–85.17]**			
*Pulmonary function*					
**Minimal chest depth (cm)**	**19.16**	**19.91**	**0.033**	3.13e -4*	2.37e -5*
	**[18.66–19.68]**	**[19.43–20.41]**			
**Maximal chest depth (cm)**	**20.50**	**21.01**	**0.146**	0.005*	3.20e -4*
	**[20.00–21.01]**	**[20.47–21.56]**			
**Forced vital capacity (FVC) (L)**	**3.32**	**4.09**	0.005*§	**0.020§**	0.008*
	**[3.02–3.63]**	**[3.78–4.40]**			
Forced expiratory volume after	2.72	3.31	0.008*§	0.177§	0.124
1 second (FEV1)(L)	[2.45–3.00]	[3.01–3.61]			
Peak expiratory flow (PEF) (L/min)	6.09	6.32	0.747§	0.638§	0.962
	[5.28–6.92]	[5.39–7.34]			
*Circulatory system*					
**Haemoglobin concentration (g/dL)**	**12.63**	**14.45**	1.05e -4*	3.51e -4*	-
	**[12.07–13.20]**	**[13.84–15.06]**			
Haemoglobin concentration** **(g/dL)**	**14.81**	**16.34**	4.88e -5§*	3.36e -4§*	-
	**[14.31–15.33]**	**[15.98–16.72]**			
Systolic pressure (mmHg)	131.76	123.94	0.071	0.245	-
	[126.14–137.23]	[117.48–129.93]			
Diastolic pressure (mmHg)	85.29	81.11	0.156	0.233	-
	[82.33–88.26]	[77.21–84.99]			
Heart rate (bpm)	67.77	64.38	0.042	0.057	-
	[65.49–70.09]	[61.67–67.2]			

Significant results are in bold.

^a^: p-value of the comparison between PNG lowlanders and PNG highlanders for raw measurements.

^b^: p-value of the comparison between PNG lowlanders and PNG highlanders for age and sex residuals.

^c^: p-value of the comparison between PNG lowlanders and PNG highlanders for age, sex and height residuals.

*: Significant Mann-Whitney U test with Bonferroni correction for 5 multiple tests (adjusted p-value = 0.01).

§: data corrected to avoid ties.

**: People suffering from anaemia were removed following the World Health Organization (WHO) standard cut-offs (exclusion of non-pregnant lowlander women < 12g/dl, lowlander men <13g/dl, exclusion of non-pregnant highlander women < 13.3g/dl, highlander men <14.3g/dl) [69].

### Body proportions

PNG highlanders have significantly shorter statures (mean: 164.6 cm [95% CI 163.21–166.03]) than PNG lowlanders (mean: 168.8 cm [95% CI 166.84–170.72]) even when corrected for age and sex (p-value = 0.001) (Table 1, S2 and S5 Figs). Our result agrees with previous studies on stature differences between highlander and lowlander groups in PNG [70], a trend also present in Tibetan, Andean, and Ethiopian populations [21, 26, 33, 71] (Table 2), although some studies found exceptions in the latter two populations [28, 51] (Table 2). It has been hypothesised that a shorter stature at high altitude might be due to the limited oxygen availability that would favour an oxygen transport system to organs at the expense of growth [47, 71]. However, the absence of association between blood oxygen saturation and height in Andean populations would indicate that it might not be the only explanation [32, 33]. Height has a complex biological pathway [72] and differences between highlanders and lowlanders could also be influenced by differences in diet and lifestyle [33, 37, 38]. Further studies would be needed to explore the mechanisms involved in height determination, but our data from PNG suggest that there is a tendency for highlanders to be of shorter stature compared with lowlanders.

**Table 2:** Variation in phenotypic traits between highlander and lowlander native groups.

Phenotypes	Papuans	Andeans	Tibetans	Ethiopians
Height	HL < LL	HL < LL [71, 73, 74]/HL = LL [51]	HL < LL [33]	HL < LL [26] / HL = LL[28]
Weight	HL = LL	HL < LL [21, 51, 75]	HL < LL [76]	HL < LL [26]/ HL > LL [28]
BMI	HL = LL	HL < LL[21, 51, 75]	HL < LL [76]	HL < LL [26]
Waist circumference	HL < LL	HL < LL [75, 77]	HL < LL [76]	NA
Chest size	HL > LL	HL > LL [21, 22, 51]	HL > LL [78]	HL > LL [28]
FVC	HL > LL	HL > LL [52, 79]	HL > LL [80]	HL > LL [28]
FEV1	HL = LL	HL > LL [52, 79]	HL > LL [80]	HL > LL [28]
PEF	HL = LL	HL > LL [52]	NA	NA
Haemoglobin concentration	HL > LL	HL > LL [81–83]	HL ≈ LL [81–84]	HL = LL [29, 31]/HL > LL [25–27]
Systole	HL = LL	HL < LL [74, 85]	HL < LL [86, 87] /HL > LL[88, 89]	HL > LL [90]
Diastole	HL = LL	HL <LL [74, 85]	HL < LL[86]/HL > LL [88, 89, 91]	HL < LL[31] / HL > LL [90]
Heart rate	HL = LL	HL = LL [51]	HL = LL [92]	HL < LL [26]

HL = highlanders LL = lowlanders*.

*:In order to condense the main comparisons of a diverse literature in this table, we used the word “lowlanders” to refer to the control group living at low altitude that studies compare with Andean, Tibetan or Ethiopian highlanders. Nonetheless, lowlander control groups may be heterogenous and vary between studies. Tibetan highlanders are traditionally compared to Han Chinese [84] but in some studies they have been compared to lowlanders from the United States of America [82]. Andeans and Ethiopians highlanders have also been compared to lowlanders from different ethnicities [25, 29, 51, 73], sometimes ethnically similar to the studied highlander groups [21, 27, 71]. The divergent result between studies may be explained by this difference in the lowlander group used.

Waist circumference is also significantly smaller in PNG highlanders (mean: 83.54 cm [95% CI 81.99–85.17]) than in PNG lowlanders (mean: 91.77 cm [95% CI 89.44–94.16]), even when corrected for age, sex and height (p-value = 0.002) (Table 1, S2, S5 and S8 Figs). Reduced waist circumference with altitude has also been observed in Tibetans and Andeans [75–77] (Table 2). Waist circumference–used as a proxy for abdominal obesity [93]–is associated with less abdominal fat and fewer cardiovascular diseases [94, 95]. It is hypothesised that the detrimental impact of obesity on respiration decreases the fitness of individuals living at high altitude [96]. Another possibility is that hypoxic conditions at high altitude reduce individual energy intake, caused by physiological and/or behavioural changes [97]. Our results show that PNG highlanders could have acquired specific biological adaptations to possibly reduce the accumulation of fat at high altitude.

Surprisingly, there is no significant difference in weight and BMI between PNG highlanders and lowlanders (p-value>0.05; Table 1). This contrasts with results obtained in other high-altitude populations for which the weight and BMI of highlanders are lower than for lowlanders [21, 26, 51, 75, 76] (Table 2). Among Tibetans, this difference was linked to metabolic adaptations counterbalancing oxidative hypoxia and diminishing the impact on basal organism energy production [98]. We note that our data show a similar tendency since the average weight for PNG lowlanders (mean: 68.46 kg [95% CI 65.86–71.15]) is higher than for PNG highlanders (mean: 64.82 kg [95% CI 63.21–66.53]), but the difference is not statistically significant (Table 1, S2, S5 and S8 Figs). This result was also found in previous studies showing no significant difference between the weight of PNG highlanders and lowlanders [70]. Our results suggest that the reduced weight and BMI observed in most highlander global populations might be mitigated in PNG by an external factor, possibly cultural. One hypothesis is that agricultural practices have been present in the highlands for at least 9,000 years [1], six millennia before the emergence in the lowlands following the arrival of Austronesian groups [2]. Agricultural practices have created prominent differences in the diet of individuals between the two regions, which are still apparent today. The diet of highlander groups is based mainly on cultivated plants and resources from the tropical altitude forest, while the diet of lowlander groups relies mainly on coastal resources and hunting [2]. A specific and more diverse diet in PNG highlanders could explain the reduced difference of weight and BMI with PNG lowlanders.

Cultural adaptation has certainly influenced body proportions especially weight, in PNG highlanders, but our data clearly suggest that these traits may be generally adapted to altitude in PNG populations.

### Pulmonary function

Measurements of chest depth during respiration significantly differ between PNG lowlanders and highlanders when corrected for age, sex and height (p-value_*depth min*_ = 2.37e -5, p-value_*depth max*_ = 3.20e -4). Highlanders have larger average values for minimal and maximal chest depths (mean minimum chest depth: 19.91 cm [95% CI 19.43–20.41]; mean maximum chest depth: 21.01 cm [95% CI 20.47–21.56]) compared with lowlanders (mean minimum chest depth: 19.16 cm [95% CI 18.66–19.68]; mean maximum chest depth: 20.50 cm [95% CI 20.00–21.01]) (Table 1, S3, S6 and S9 Figs). A larger chest size has been associated with a greater lung capacity in Andeans and Tibetans, and also potentially Ethiopian highlanders [21, 22, 28, 52, 61, 78, 99] (Table 2). The combination of a larger chest size with a smaller stature in highlanders has been related to a larger ventilatory capacity to body mass [100], suggesting the possibility of similar adaptive responses in PNG highlanders.

We tested pulmonary function of PNG highlanders and lowlanders. PNG highlanders show a significant higher forced vital capacity (FVC) than PNG lowlanders (mean highlanders: 4.09 L [95% CI 3.78–4.40]; mean lowlanders: 3.32 L [95% CI 3.02–3.63]) even after correction for age, sex and height (p-value = 0.008) (Table 1, S3, S6 and S9 Figs).

We also detected a difference in forced expiratory volumes (FEV1) between PNG highlanders (mean: 3.31L [95% CI 3.01–3.61]) and lowlanders (mean: 2.72L [95% CI 2.45–3.00]), although this was not significant after correction (Table 1, S3, S6 and S9 Figs). Higher spirometer measurements have been previously reported in other PNG highlander and lowlander populations [43, 44] and among Andean [52, 79], Tibetan [80] and Ethiopian highlanders [28] (Table 2). Our results confirm that–in agreement with these three other altitude populations—PNG highlanders tend to have a larger pulmonary volume than lowlanders. At 2,500 m a.s.l., the average altitude of studied PNG highlanders, the oxygen partial pressure is only around 70% of its level at sea level [101]. An adaptive strategy is to increase the quantity of inspired air in the lungs to compensate for the lower oxygen availability at high altitude. Despite being located at the fringe of what is recognised as high altitude [19], PNG highlanders clearly show a combination of specific phenotypes that indicate adapted pulmonary function.

### Circulatory system

Haemoglobin concentration is significantly different between PNG highlanders and lowlanders, even after correction for age and sex (p-value = 3.51e -4). PNG highlanders have a higher mean haemoglobin concentration (mean: 14.45 g/dl [95% CI 13.84–15.06]) compared with PNG lowlanders (mean: 12.63 g/dl [95% CI 12.07–13.20]) (Table 1, S4 and S7 Figs). This higher haemoglobin concentration in PNG highlanders has already been shown in a previous study on the prevalence of anaemia in PNG [45]. In addition to altitude, malaria is an important cofactor in the haemoglobin concentration difference between PNG lowlanders and highlanders [102, 103]. In order to minimise the impact of malaria prevalence differences between PNG lowlands and highlands, we removed people suffering from anaemia from the haemoglobin comparison analysis, following the World Health Organization (WHO) standard cut-offs. These cut-offs have been adjusted to limit the underestimation of anaemia among PNG highlanders following the altitude adjustment suggested by WHO (exclusion of non-pregnant lowlander women < 12 g/dl, lowlander men <13 g/dl, exclusion of non-pregnant highlander women < 13.3 g/dl, highlander men <14.3 g/dl) [69, 104]. This filter did not change the significant difference of haemoglobin concentration between PNG highlanders (mean: 16.34 g/dl [95% CI 15.98–16.72]) and lowlanders (mean: 14.81 g/dl [95% CI 14.31–15.33]) (Table 1, S4 and S7 Figs). Malaria could be a confounding variable in our data, but PNG highlanders show a significant difference in a phenotypic trait also found in other high-altitude populations such as Andeans [23, 81, 83], and probably Ethiopian highlanders [28, 29]. In contrast, high-altitude Tibetans display a reduced haemoglobin concentration [23, 42, 81, 84] (Table 2). A recent study on Andeans and Tibetans suggested that high-altitude selection does not directly impact haemoglobin concentration but rather haemoglobin mass and plasma volume [24]. High haemoglobin concentration is also observed in acclimatised lowlanders living at high altitude and is associated with an increase in blood viscosity, which is maladaptive and can cause Chronic Mountain Sickness [105, 106]. Higher haemoglobin concentration in PNG highlanders is a phenotype typically observed at high altitude that may help to counteract a lower oxygen saturation due to hypoxia. Further studies will be needed to determine whether this is an acclimatization response or whether PNG highlanders may have experienced an adaptive trajectory.

We measured three phenotypic traits related to heart activity: heart rate, diastolic and systolic blood pressure. Heart rate is not significantly different between PNG highlanders (mean: 67.77 bpm [95% CI 65.49–70.09]) and lowlanders (mean: 64.38 bpm [95% CI 61.67–67.2]) (Table 1, S4 and S7 Figs). Similar results were found in Tibetans and Andeans [52, 92] (Table 2). This non-significance is interesting because lowlander individuals have an increased heart rate during short-term stays at altitude, which has been associated with acute mountain sickness [107, 108]. Among Tibetans, absence of heart rate increase at high altitude has been suggested to be an adaptation to acute exposure to high altitude [92] that may be passed down through the generations [109]. In addition, a lower heart rate was also linked to a greater haemoglobin mass in Tibetans [24]. While we cannot deny the impact of other factors, including physical activity and diet, on the heart rate of PNG highlanders, the role of a lower heart rate in the adaptation to acute exposure to high altitude should be considered.

There is no significant difference in diastolic and systolic blood pressures between PNG highlanders and lowlanders (Table 1, S4 and S7 Figs). The blood pressure of Andean highlanders is lower than Andean lowlanders [74, 85], while there are uncertainties regarding Tibetans [86, 88] and Ethiopians [31, 90] (Table 2). A higher prevalence of hypertension was shown in acclimatised lowlanders at high altitude [110], which would suggest that the observed blood pressure in PNG highlanders is potentially a protective trait. The absence of an increase in heart rate and blood pressure in PNG highlanders at altitude may indicate that their circulatory system is adapted to their environment.

We found significant differences among PNG highlanders for phenotypes related to body proportions, pulmonary function and circulatory system (Table 1, S2–S9 Figs).

Our study raises a major question on the adaptive processes to altitude in Oceania. Our data might be influenced by phenotypic changes caused by long-term stays at high altitude independent of ancestry (i.e., acclimatization), due to adaptation to environmental pressures other than hypoxia (e.g., cold), population differentiation following genetic drift, or lifestyle differences (e.g., diet, training state). However, they could also reveal heritable, genetically coded traits (i.e., selection) as has been observed in other intermediate populations from the Andean plateaus and Ethiopian highlands [30, 52].

This latter hypothesis is particularly interesting since PNG populations have settled in the highlands for at least 20,000 years [3]. These populations are some of the oldest continuous settlements at high altitude in the world [111, 112]. We previously found that the demographic history of PNG populations is marked by relatively low population mobility and strong genetic differentiation among groups, especially in the highlands [5]. This might have favoured the emergence of highly specific adaptations to altitude.

Many of these traits were also observed in other high-altitude populations worldwide, suggesting that altitude acts on the same biological pathways, independent of latitude (Table 2). Convergent adaptations to altitude were found in the HIF-pathway [25, 39]. However, previous studies have found that parts of adaptive strategies can be population-specific [101]. While some of the candidates genes involved in the HIF pathway are common to several high-altitude populations (e.g., EGLN1 in both Tibetan and Andean highlanders) some are exclusive to a specific population (e.g., PRKAA1 in Andean highlanders) [113]. Finally, some candidate genes are part of totally different pathways (e.g., nitric oxide pathway [52]). Whether PNG highlanders share similar genetic adaptions with other high-altitude populations and/or exclusive ones due to 50,000 years of isolation from the rest of the world remains to be explored.

Another hypothesis would be an adaptative introgression from Denisovan knowing that Papuans carry the highest proportion of Denisovan introgressions and that low haemoglobin concentration at high altitude in Tibetans is potentially inherited from Denisovan [42]. Currently PNG genomes are only available for lowlanders and groups from moderate altitude (~1,500m a.s.l.) [114–118] which would explain the absence of detected adaptative introgression to high altitude. Future genomic analyses on the high-altitude PNG communities studied in this work might be of great interest.

## Conclusion

Our study shows that high altitude is a major factor contributing to the phenotypic variation observed in PNG. PNG highlanders tend to have a shorter stature, a larger chest depth and pulmonary volume, and a higher haemoglobin concentration compared with PNG lowlanders. The phenotypic differences observed in this study between lowlanders and highlanders, may be considered as preliminary data indicating mild positive selection in response to mild selective pressure for hypoxia. This hypothesis will request further physiological and genetic studies to be confirmed. The long-isolated history of PNG populations may have favoured the independent emergence of biological traits adapted to their environment. The driving force is probably multifactorial, including cultural, environmental, and genetic aspects. Although human biology is plastic, allowing individuals to temporarily adapt to new ecological contexts, altitude is a major selective pressure on the genomes of human populations. Given their unique genetic diversity, genomic data from PNG highlanders have the potential to shed new light on an exceptional chapter of the history of human biological adaptation.

## Supporting information

S1 Fig Correlation matrix between the studied phenotype measurements. Spearman correlation was used. Only significant correlations (p-val <0.05) are showed. Selected 5 groups of correlated phenotypes are enclosed in red boxes.
(TIF)

S2 Fig Violin plots comparing the body proportions measurements between PNG highlanders and PNG lowlanders. P-value are given for Mann-Whitney U test comparison between PNG lowlanders and PNG highlanders for raw measurements.*: Significant Mann-Whitney U test with Bonferroni correction for 5 multiple tests (adjusted p-value = 0.01)
(TIF)

S3 Fig Violin plots comparing the pulmonary function measurements between PNG highlanders and PNG lowlanders. P-value are given for Mann-Whitney U test comparison between PNG lowlanders and PNG highlanders for raw measurements. *: Significant Mann-Whitney U test with Bonferroni correction for 5 multiple tests (adjusted p-value = 0.01). §: data corrected to avoid ties.
(TIF)

S4 Fig Violin plots comparing the circulatory system measurements between PNG highlanders and PNG lowlanders. P-value are given for Mann-Whitney U test comparison between PNG lowlanders and PNG highlanders for raw measurements. *: Significant Mann-Whitney U test with Bonferroni correction for 5 multiple tests (adjusted p-value = 0.01). §: data corrected to avoid ties. **: People suffering from anaemia were removed from these violin plots, following the World Health Organization (WHO) standard cut-offs (exclusion of non-pregnant lowlander women < 12g/dl, lowlander men <13g/dl, exclusion of non-pregnant highlander women < 13.3g/dl, highlander men <14.3g/dl))[69].
(TIF)

S5 Fig Violin plots comparing age and sex residuals for body proportions measurements between PNG highlanders and PNG lowlanders. P-value are given for Mann-Whitney U test comparison between PNG lowlanders and PNG highlanders for age and sex residuals. *: Significant Mann-Whitney U test with Bonferroni correction for 5 multiple tests (adjusted p-value = 0.01)
(TIF)

S6 Fig Violin plots comparing age and sex residuals for pulmonary function between PNG highlanders and PNG lowlanders. P-value are given for Mann-Whitney U test comparison between PNG lowlanders and PNG highlanders for age and sex residuals. *: Significant Mann-Whitney U test with Bonferroni correction for 5 multiple tests (adjusted p-value = 0.01). §: data corrected to avoid ties.
(TIF)

S7 Fig Violin plots comparing age and sex residuals for circulatory system measurements between PNG highlanders and PNG lowlanders. P-value are given for Mann-Whitney U test comparison between PNG lowlanders and PNG highlanders for age and sex residuals. *: Significant Mann-Whitney U test with Bonferroni correction for 5 multiple tests (adjusted p-value = 0.01). §: data corrected to avoid ties. **: People suffering from anaemia were removed from these violin plots, following the World Health Organization (WHO) standard cut-offs (exclusion of non-pregnant lowlander women < 12g/dl, lowlander men <13g/dl, exclusion of non-pregnant highlander women < 13.3g/dl, highlander men <14.3g/dl))[69].
(TIF)

S8 Fig Violin plots comparing age, sex and height residuals for body proportions measurements between PNG highlanders and PNG lowlanders. P-value are given for Mann-Whitney U test comparison between PNG lowlanders and PNG highlanders for age and sex residuals. *: Significant Mann-Whitney U test with Bonferroni correction for 5 multiple tests (adjusted p-value = 0.01).
(TIF)

S9 Fig Violin plots comparing age, sex and height residuals for pulmonary function measurements between PNG highlanders and PNG lowlanders. P-value are given for Mann-Whitney U test comparison between PNG lowlanders and PNG highlanders for age and sex residuals. *: Significant Mann-Whitney U test with Bonferroni correction for 5 multiple tests (adjusted p-value = 0.01).
(TIF)

S1 Table Number of phenotypic measurements for PNG highlanders and PNG lowlanders. (PDF)

S2 Table Raw measurements for the 13 studied phenotypes. (XLSX)
