# Supplementary figures and images for: Phenotypic differences between highlanders and lowlanders in Papua New Guinea
Source: PLoS One. 2021 Jul 21;16(7):e0253921. doi: 10.1371/journal.pone.0253921 (PMC8294550; doi:10.1371/journal.pone.0253921)

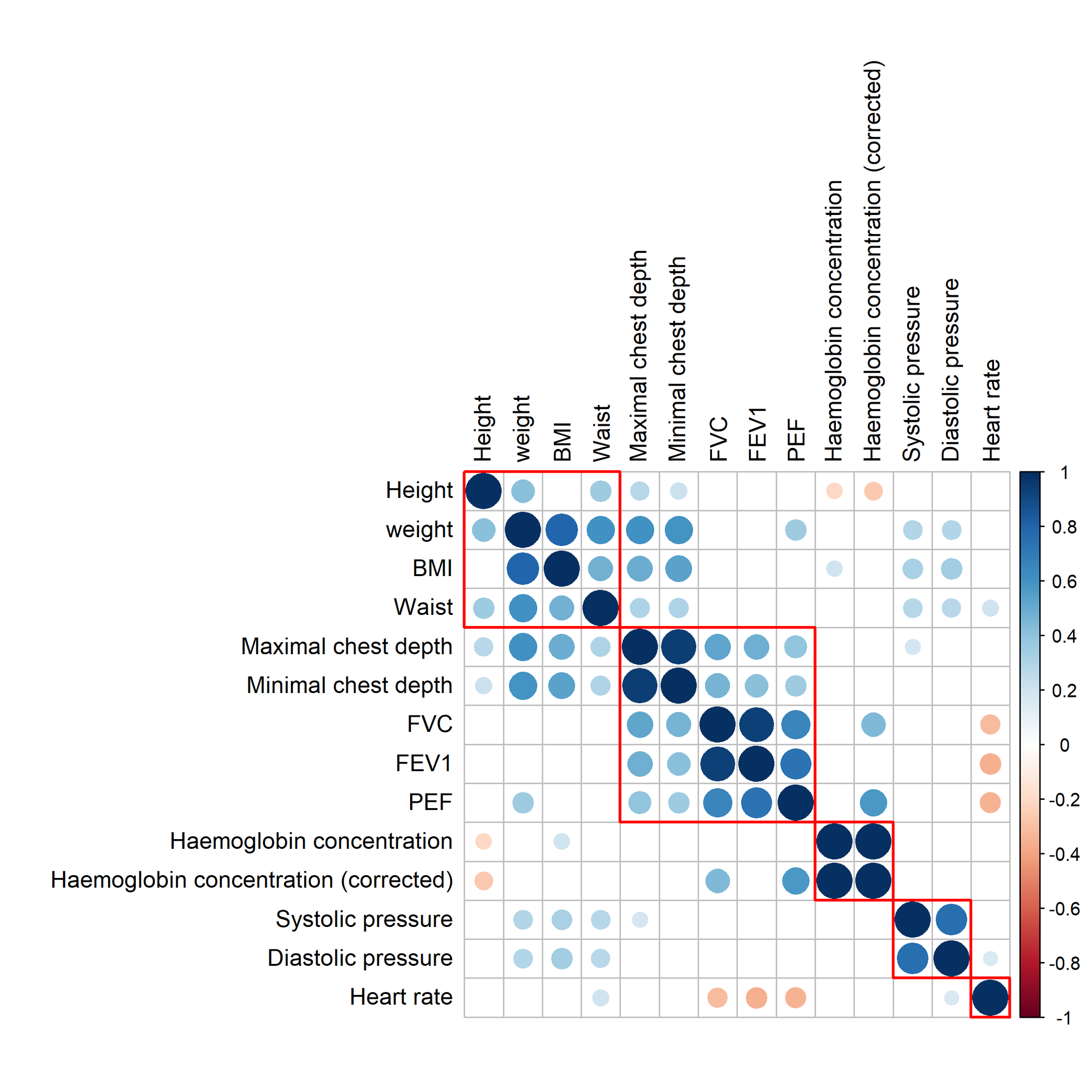

Supplement: S1 Fig — Spearman correlation was used. Only significant correlations (p-val <0.05) are showed. Selected 5 groups of correlated phenotypes are enclosed in red boxes. (TIF) [file pone.0253921.s001.tif]

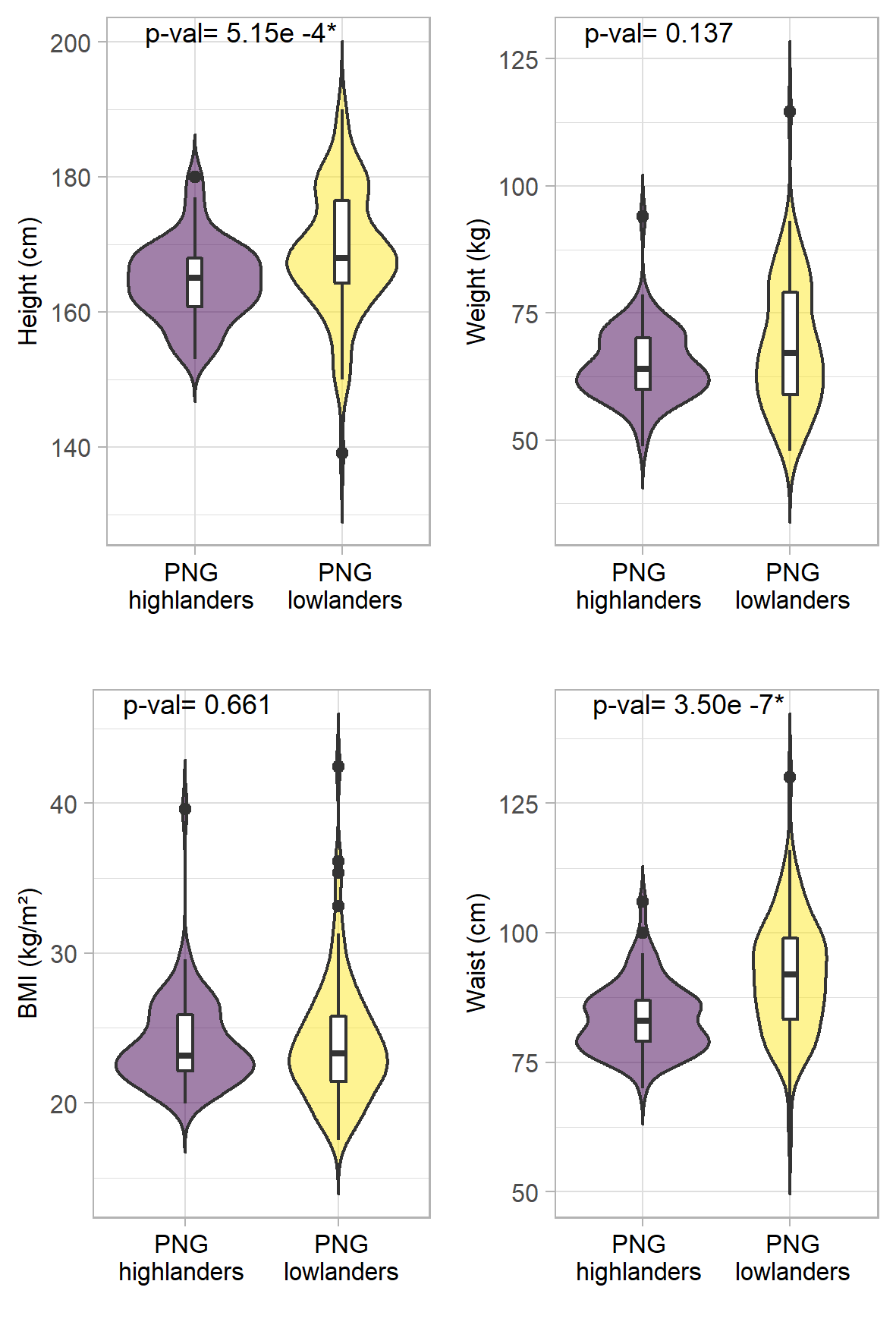

Supplement: S2 Fig — P-value are given for Mann-Whitney U test comparison between PNG lowlanders and PNG highlanders for raw measurements.*: Significant Mann-Whitney U test with Bonferroni correction for 5 multiple tests (adjusted p-value = 0.01) (TIF) [file pone.0253921.s002.tif]

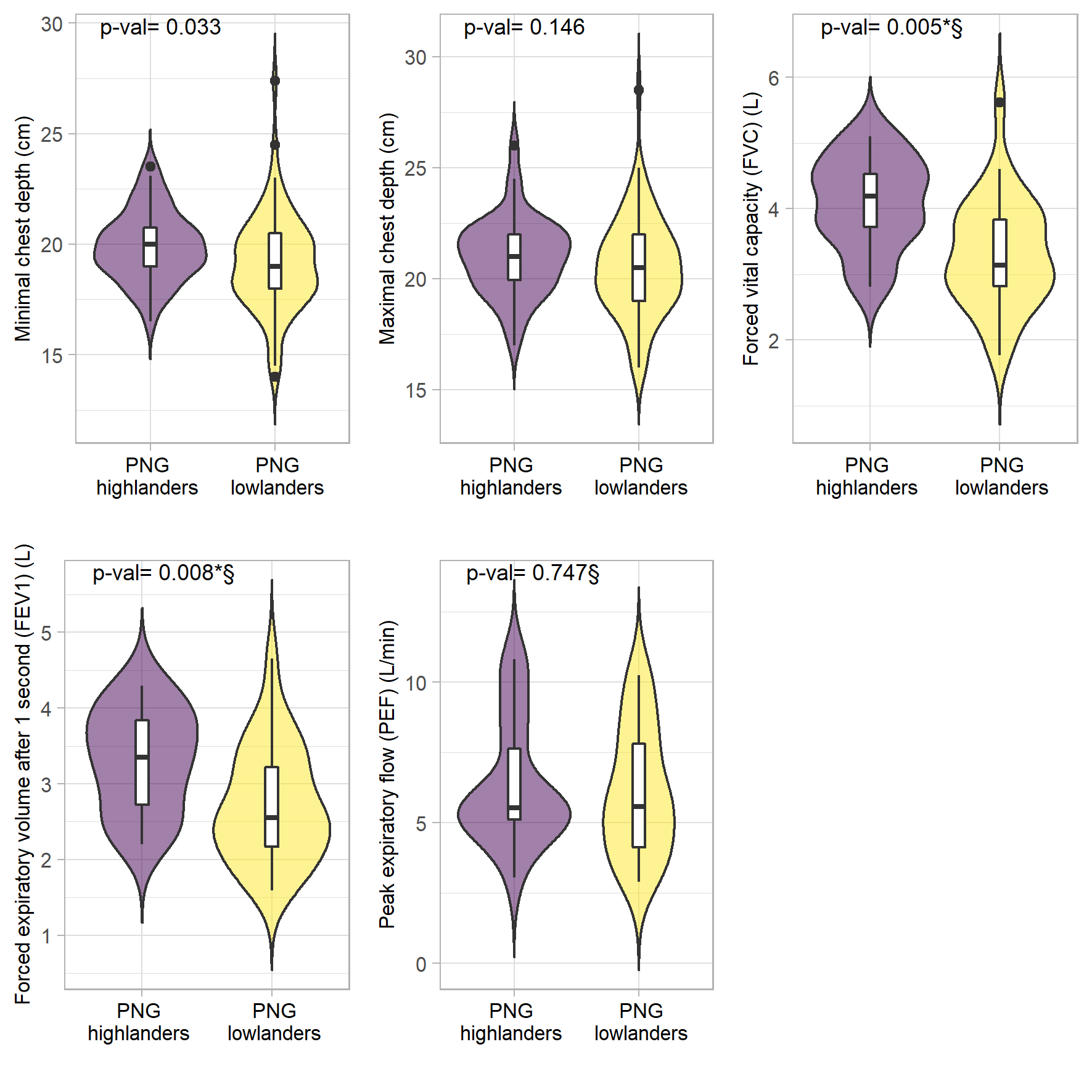

Supplement: S3 Fig — P-value are given for Mann-Whitney U test comparison between PNG lowlanders and PNG highlanders for raw measurements. *: Significant Mann-Whitney U test with Bonferroni correction for 5 multiple tests (adjusted p-value = 0.01). §: data corrected to avoid ties. (TIF) [file pone.0253921.s003.tif]

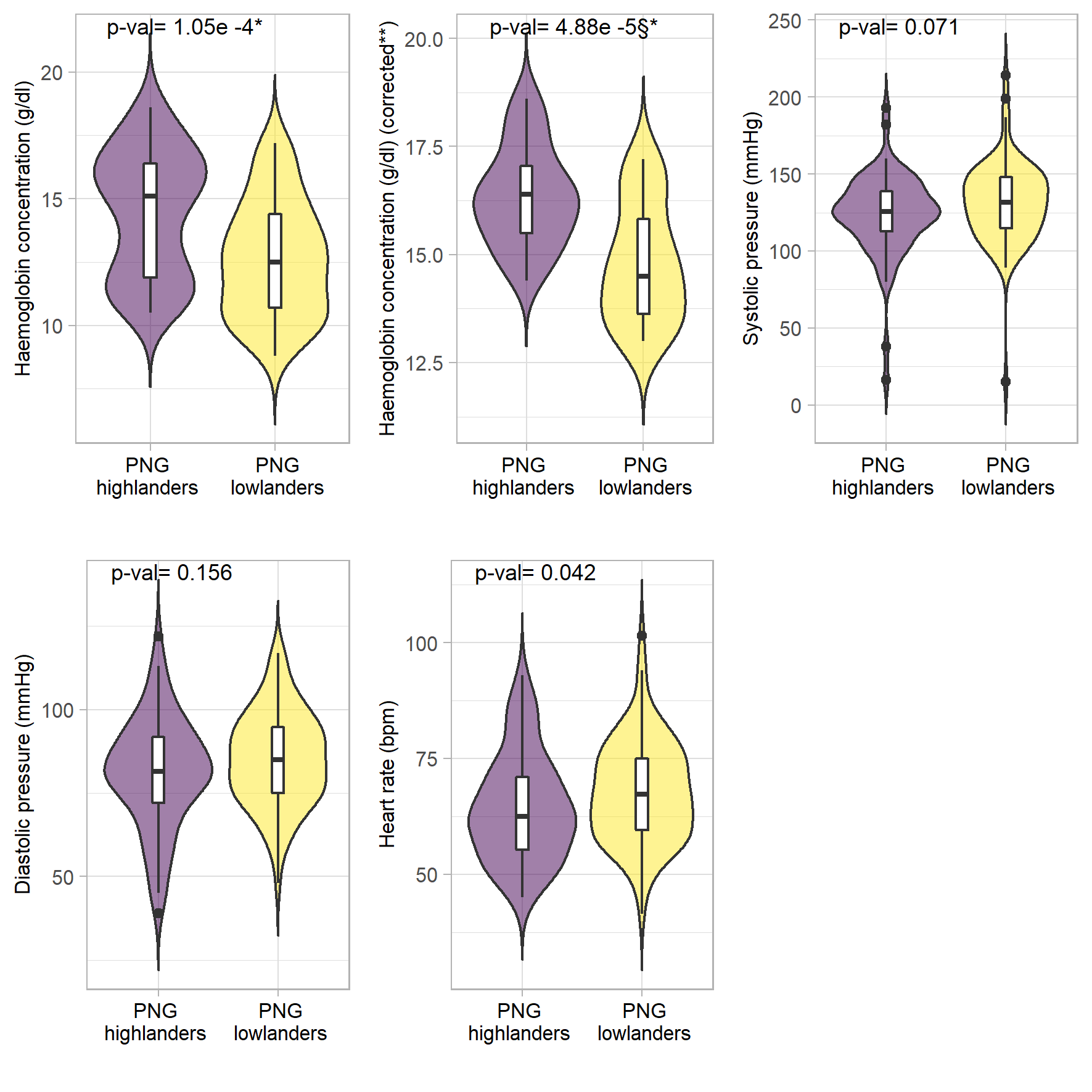

Supplement: S4 Fig — P-value are given for Mann-Whitney U test comparison between PNG lowlanders and PNG highlanders for raw measurements. *: Significant Mann-Whitney U test with Bonferroni correction for 5 multiple tests (adjusted p-value = 0.01). §: data corrected to avoid ties. **: People suffering from anaemia were removed from these violin plots, following the World Health Organization (WHO) standard cut-offs (exclusion of non-pregnant lowlander women < 12g/dl, lowlander men <13g/dl, exclusion of non-pregnant highlander women < 13.3g/dl, highlander men <14.3g/dl))[69]. (TIF) [file pone.0253921.s004.tif]

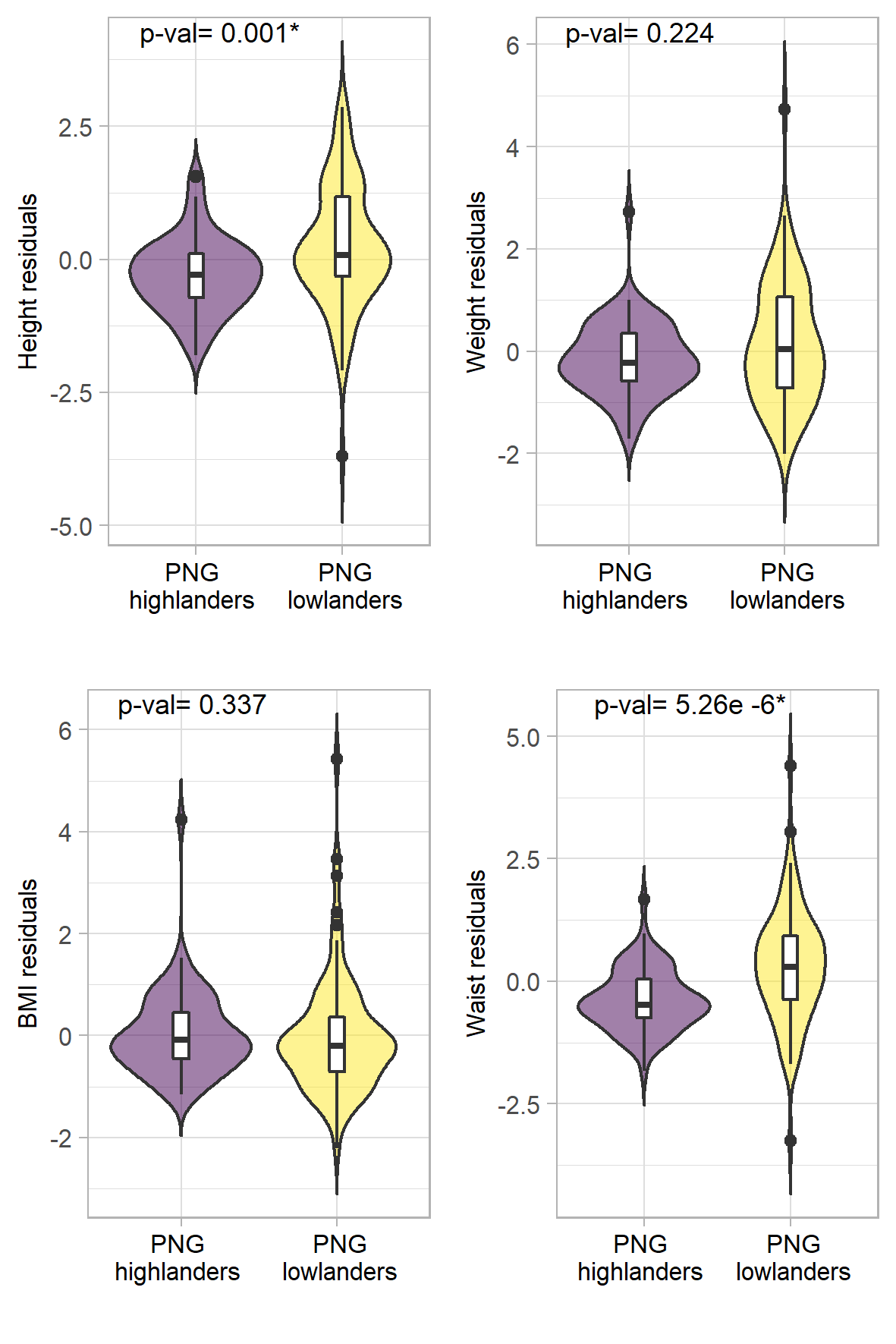

Supplement: S5 Fig — P-value are given for Mann-Whitney U test comparison between PNG lowlanders and PNG highlanders for age and sex residuals. *: Significant Mann-Whitney U test with Bonferroni correction for 5 multiple tests (adjusted p-value = 0.01) (TIF) [file pone.0253921.s005.tif]

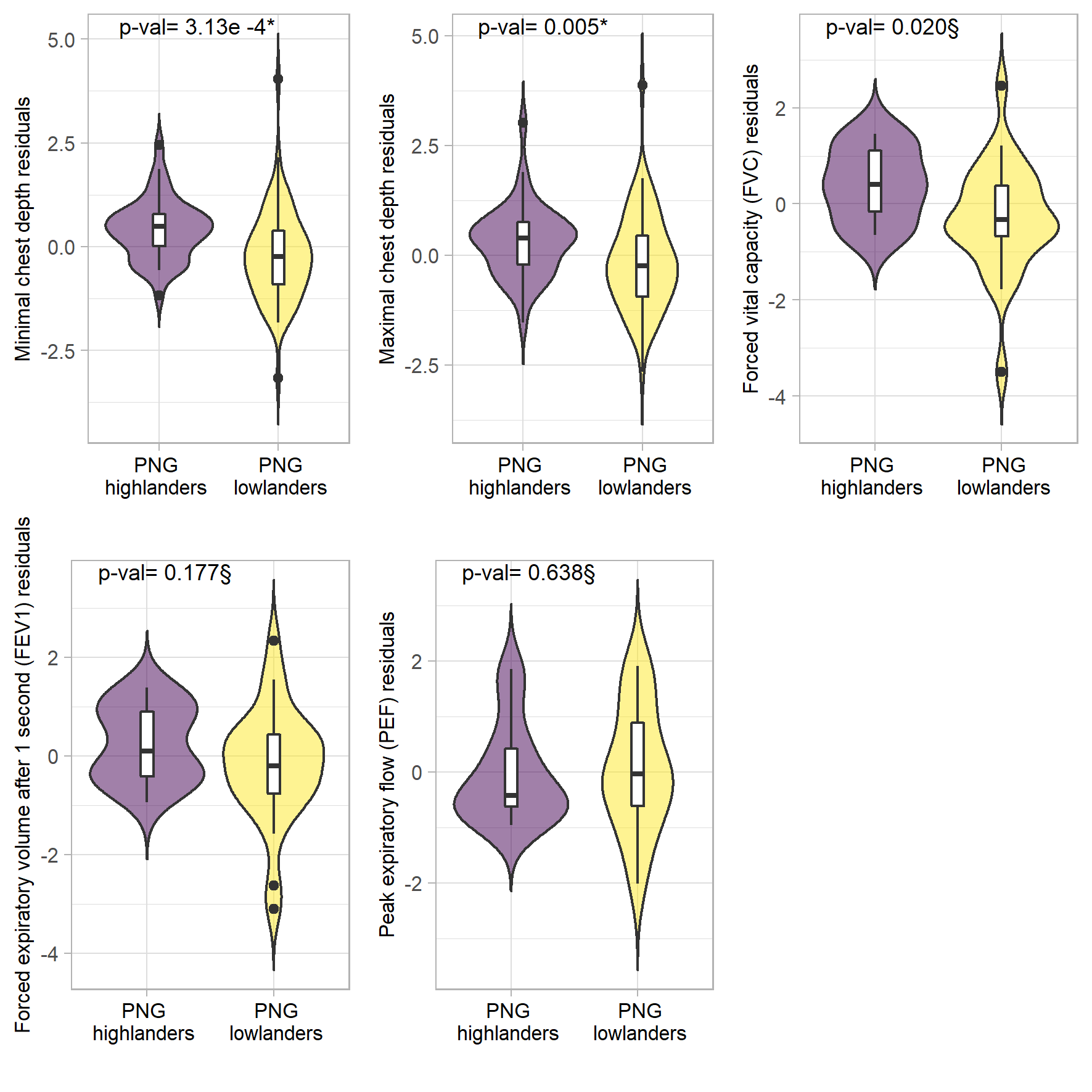

Supplement: S6 Fig — P-value are given for Mann-Whitney U test comparison between PNG lowlanders and PNG highlanders for age and sex residuals. *: Significant Mann-Whitney U test with Bonferroni correction for 5 multiple tests (adjusted p-value = 0.01). §: data corrected to avoid ties. (TIF) [file pone.0253921.s006.tif]

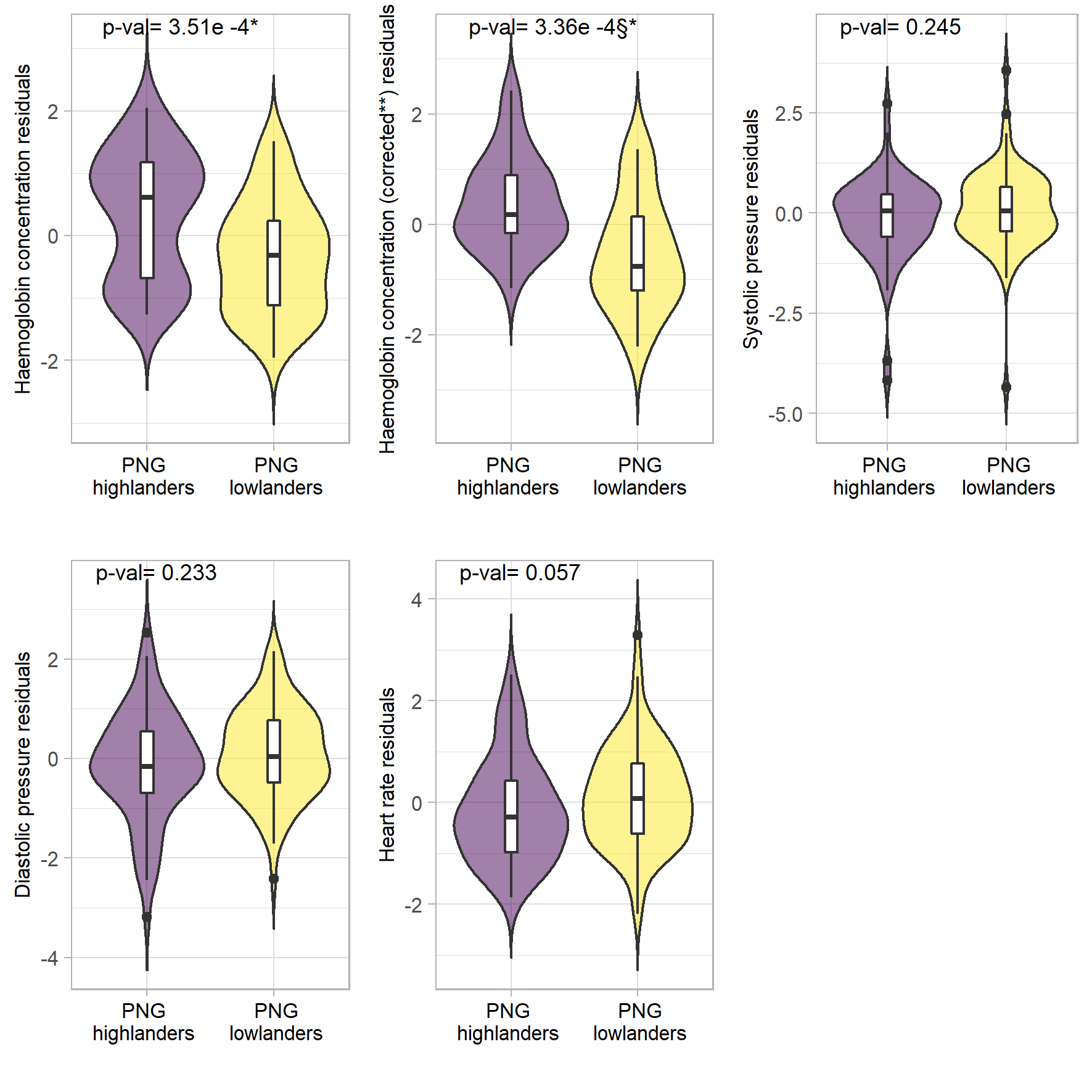

Supplement: S7 Fig — P-value are given for Mann-Whitney U test comparison between PNG lowlanders and PNG highlanders for age and sex residuals. *: Significant Mann-Whitney U test with Bonferroni correction for 5 multiple tests (adjusted p-value = 0.01). §: data corrected to avoid ties. **: People suffering from anaemia were removed from these violin plots, following the World Health Organization (WHO) standard cut-offs (exclusion of non-pregnant lowlander women < 12g/dl, lowlander men <13g/dl, exclusion of non-pregnant highlander women < 13.3g/dl, highlander men <14.3g/dl))[69]. (TIF) [file pone.0253921.s007.tif]

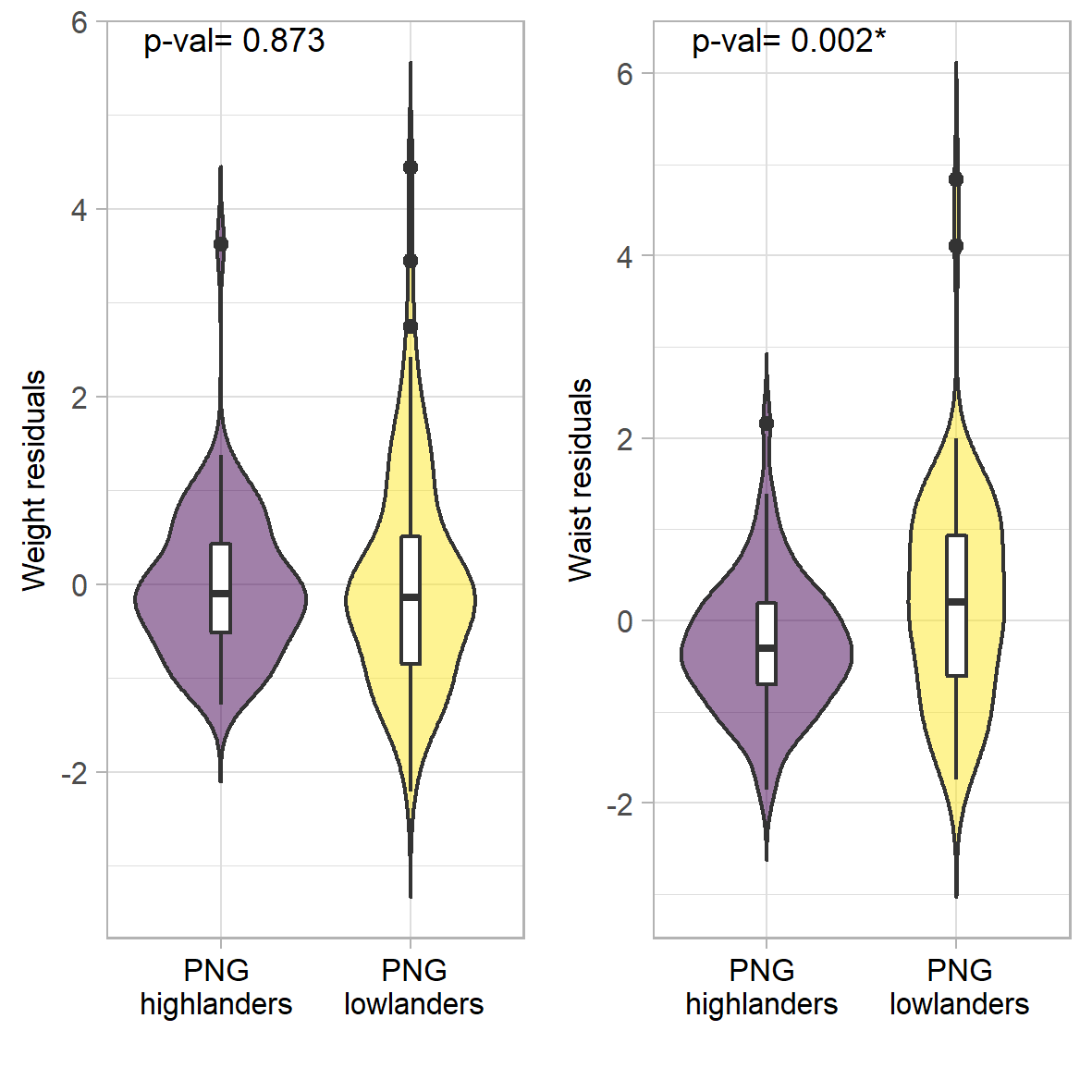

Supplement: S8 Fig — P-value are given for Mann-Whitney U test comparison between PNG lowlanders and PNG highlanders for age and sex residuals. *: Significant Mann-Whitney U test with Bonferroni correction for 5 multiple tests (adjusted p-value = 0.01). (TIF) [file pone.0253921.s008.tif]

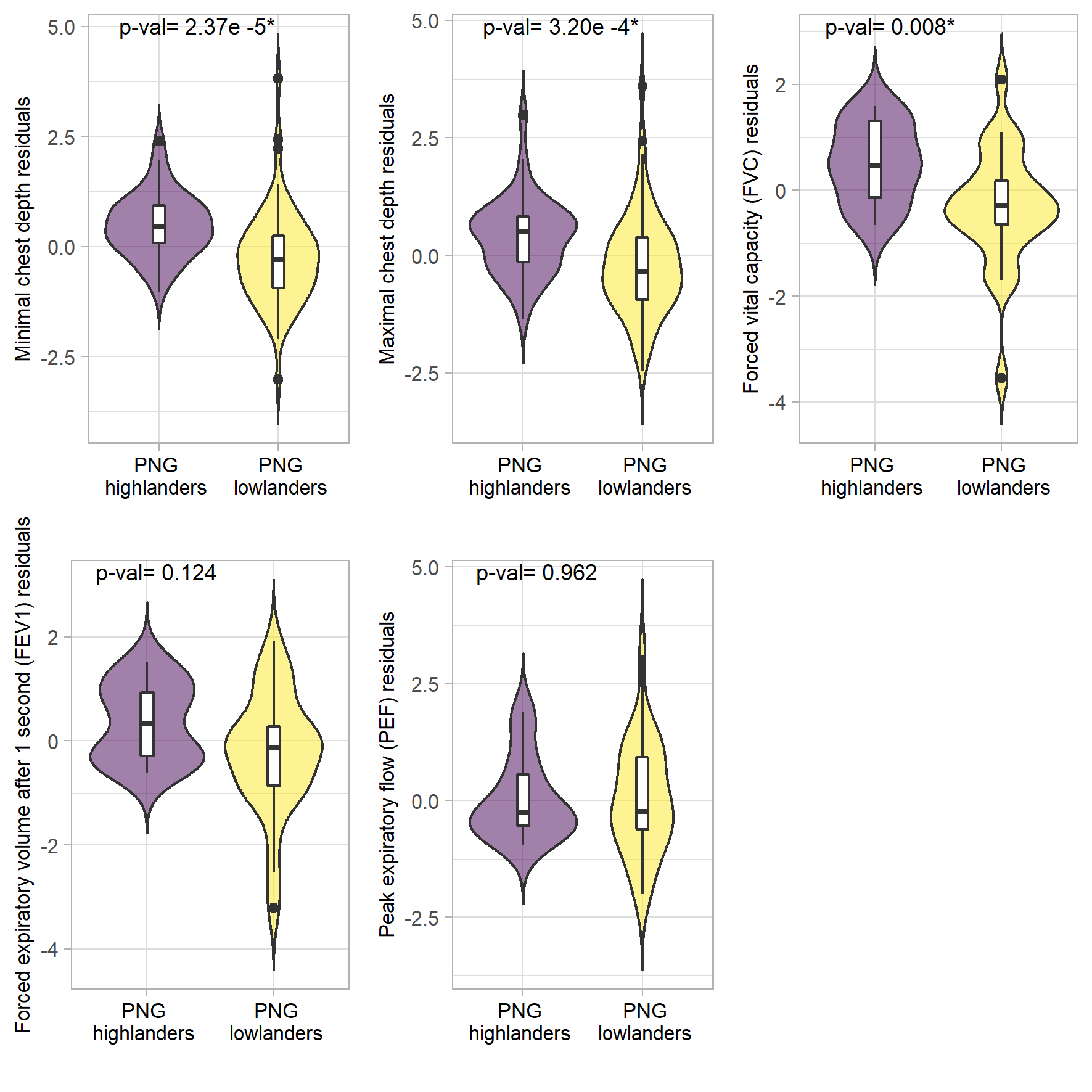

Supplement: S9 Fig — P-value are given for Mann-Whitney U test comparison between PNG lowlanders and PNG highlanders for age and sex residuals. *: Significant Mann-Whitney U test with Bonferroni correction for 5 multiple tests (adjusted p-value = 0.01). (TIF) [file pone.0253921.s009.tif]
