# Supplementary material for: Phenotypic differences between highlanders and lowlanders in Papua New Guinea
Source: PLoS One. 2021 Jul 21;16(7):e0253921. doi: 10.1371/journal.pone.0253921 (PMC8294550; doi:10.1371/journal.pone.0253921)
Supplement: S1 Table — (PDF) [file pone.0253921.s010.pdf]

S1 Table. **Number of phenotypic measurements for PNG highlanders and PNG lowlanders.**

	**PNG lowlanders**<br>N= 86	**PNG highlanders**<br>N= 70
age (years) [95%CI]	45.36 [42.26 - 48.46]	38.50 [34.31 – 42.81]
men	77	62
women	9	8
**Body proportions**		
Height	86	68
Weight	86	69
BMI	86	68
Waist circumference	86	69
**Pulmonary function**		
Minimal chest depth	73	39
Maximal chest depth	73	40
Forced vital capacity (FVC)	28	18
Forced expiratory volume after 1 second (FEV1)	28	18
Peak expiratory flow (PEF)	28	18
**Circulatory system**		
Haemoglobin concentration	61	61
Haemoglobin concentration (corrected*)	26	35
Systolic pressure	86	70
Diastolic pressure	86	70
Heart rate	86	66

*: People suffering from anaemia were removed from these violin plots, following the World Health Organization (WHO) standard cut-offs (exclusion of non-pregnant lowlander women < 12g/dl, lowlander men <13g/dl, exclusion of non-pregnant highlander women < 13.3g/dl, highlander men <14.3g/dl))[68].
